# Nutritional management of food allergies: Prevention and treatment

**DOI:** 10.3389/falgy.2022.1083669

**Published:** 2023-01-06

**Authors:** Ludovica Leone, Alessandra Mazzocchi, Laura Maffeis, Valentina De Cosmi, Carlo Agostoni

**Affiliations:** ^1^ Pediatric Unit - Foundation, IRCCS Ca' Granda - Ospedale, Maggiore, Policlinico, Milan, Italy; ^2^ Department of Clinical Sciences and Community Health, University of Milano, Milano, Italy

**Keywords:** food allergy, prevention, exclusion diet, nutritional management, dietitian

## Abstract

An individualized allergen avoidance plan is the cornerstone of the nutritional management of food allergy (FA). In pediatric age, the main objective is preventing the occurrence of acute and chronic symptoms by avoiding the offending food(s) and providing an adequate, nutritionally balanced and personalized diet at the same time. For this reason, the presence of a trained dietitian is recommended in order to meet nutritional needs of patients with FA and to provide a tailored nutritional plan, minimizing the impact of FA on quality of life and maintaining optimal growth.

## 1. Introduction

Data from the most recent literature show the prevalence of allergic disorders increasing. In particular, to date, in every ten people, one lives with a food allergy (FA) and the prevalence is higher in the pediatric population.

Being a multifactorial disease, FA is influenced by genetics, environment, and their interactions. The main risk factors involved in the expression or sensitization to FA include: sex (in particular, being male in children), race/ethnicity (the risk is higher among Asian and black children); genetics. Moreover, other potential risk factors can also contribute: atopic comorbidity, increased hygiene, microbiome, serum vitamin D levels, diet (i.e., low intake of omega-3-polyunsaturated fatty acids, reduced consumption of antioxidants), increased use of antiacids, obesity, timing of food introduction and exposure to foods in a different way from the gastrointestinal one.

In high-income countries, cow's milk, eggs, wheat, fish and shellfish, peanuts, nuts, and soy are the most frequent responsible for FA in children. Above them, some food allergies have a high rate of resolution before adolescence. Data from literature say that milk allergy resolves in up to 50% of patients by age 5–10 years, egg allergy resolves about in half of the cases by ages 2–9 years, wheat allergy in 50% of children affected by age 7 years, and soy allergy in 45% by age 6 years.

Other food allergies typically persist in childhood; peanut allergy is estimated to resolve in about 20% of patients by age 4 years, tree nut allergy in only 10% of patients. Allergy to seeds, fish, and shellfish are considered persistent (1).

Excluding nutritionally essential foods from the diet on one's own initiative may cause harm rather than benefit, especially in a young child. Proper dietary guidance should be given only on the basis of a definite diagnosis of FA through specific examinations.

A precise identification of the culprit foods is essential for an individualized management of the patient. In this perspective, diagnostic methods, like component-resolved diagnostics (CRD) and the epitope reactivity, can allow a more targeted analysis of the disease and a tailored therapeutic plan. Clinical history, skin prick test, food-allergen IgE, exclusion diets and subsequent oral food challenges are important FA diagnostic tools (2).

The oral food challenge (OFC) can be performed either open or single-blind food challenge, or better as double-blind placebo-controlled food challenge (DBPCFC). Actually, DBPCFC is still the gold standard for FA diagnosis (3) even if its execution in pediatric age can be more difficult that in adults. Moreover, OFC is a procedure that is not free from major risks (i.e., serious anaphylactic reactions) (4).

Recently, other diagnostic test *in vitro*, such as CRD and the basophil activation test (BAT) have been developed to estimate the risk of serious, life-threating reactions before OFC (5).

The diagnosis of FA makes it possible to identify which foods need to be eliminated and is essential in order to be able to devise a nutritionally balanced, individualized exclusion diet. Allergic children and parents must, in addition, be well educated in the interpretation and reading of nutrition labels in order to learn how to avoid specific and hidden allergens, all of which make it possible to prevent new allergic reactions and thus the onset of more severe symptoms.

## 2. Growth in allergic children: A wake-up call

Inadequate growth in children affected by food allergy, compared with their healthy peers, is well documented. Some studies have found lower energy intake in the diets of allergic children and different growth patterns compared with healthy children, even when nutrient intake is similar. The hypothesis for the poor growth in this pediatric population can be related not only to the high number of foods to be excluded due to allergy but also to a persistent sub-inflammatory state of the gastrointestinal mucosa. It may reduce the absorption of fuels and substrates and increase intestinal permeability and, consequently, nutrient loss (6).

In contrast, other studies point out that obesity can also occur in allergic children due to unbalanced diets and poor food choices (7).

This potential difference in growth highlights the need to make every effort to optimize nutritional intervention.

From a nutritional point of view, measuring growth in children reflects the adequacy of the diet. However, growth control must be followed by regular evaluation of other signs of deficiency. Recurrent reviews of food intake to assess possible supplementation are also essential; this highlights the importance of appropriate counseling by an expert dietitian, who can promptly and early manage nutritional problems resulting from the exclusion of specific foods.

It is well known that childhood is a period of rapid growth and that optimal and balance intake of micro and macronutrients is fundamental for optimal growth.

Weight, length, Body mass index, and head circumference are the indicators mainly used to measure growth rates in infants and children.

Nowadays, World Health Organization propose cutoff points, expressed in z-score units, to evaluate inappropriate nutritional status. The same z-score units are used in epidemiological studies, too.

## 3. Nutritional intervention

### 3.1. Prevention and treatment

The first three years of life are considered an age of opportunity and intervention. During this period of life nutritional factors, among others, may impact the risk of developing allergies through epigenetic mechanisms. Consequently, the nutritional interventions are well recognized as central players both in the prevention and treatment of food allergies. Recently, different clinical approaches emerged. The traditional approach consisted of protracted food allergen avoidance.

This practice contributed to a dramatic increase of anaphylaxis.

On the contrary, the new approach suggests to introduce complementary foods in the diet between 4 and 6 months of life, even in infants at higher allergic risk. Others nutritional strategies potentially able to educate the immune system towards oral tolerance are the approach of gradual introduction of small quantities of foods allergens in a less allergenic form to higher and more allergenic quantities of food allergens.

Delaying food introduction does not protect against allergy development. Excessive delay in the introduction of allergenic foods could increase the risk of sensitization, instead. Two randomized clinical trials showed that the early introduction of peanuts and other food allergens can reduce the risk of FA (8, 9). More recently, two population-based studies including more than 7,000 participants in Melbourne, Australia, examined the impact of earlier peanut introduction on the frequency of peanut allergy. Peanut introduction in the first year of life increased from 28 to 88% over this time period. However, there was a slight diminution in peanut allergy prevalence, from 3.1 to 2.6%. This result highlighted that other interventions should be done in order to prevent peanut allergy in the general population (10).

This led to the hypothesis that similar mechanisms of defense may be present for other food as for peanut and that different “windows of opportunity” may exist for each food. Additional approaches will be necessary to prevent food allergies in infants who fail to benefit from early allergen introduction.

The European Academy of Allergology and Clinical Immunology (EAACI) revised its guidelines for the prevention of FA in infants and children in 2020 (11).

The main changes from the 2014 version include the suggestion to introduce peanuts and well-cooked eggs during complementary feeding and to avoid, for the first seven days of life, supplementation with cow's milk. The main recommendations are listed in Table 1.

**Table 1:** Nutritional strategies for the prevention of food allergy.

✓ *Exclusive breastfeeding for 4–6 months. Introduction of complementary diet from 4 to 6 months of age.*
✓ *Consider infants at high risk for food allergy when they have a personal history of atopy or a first-degree relative (at least one parent or sibling) with an atopic condition (such as asthma, allergic rhinitis, food allergy, or eczema).*
✓ *Promote and support breastfeeding for up to 2 years and beyond, regardless of issues pertaining to food allergy prevention.*
✓ *There is still insufficient evidence to recommend modifying the maternal diet to prevent food allergy.*
✓ *Breastfeeding of all infants is preferable, but when a breastmilk substitute is needed, professionals could help families consider the best possible alternative for a family's individual circumstances. The options discussed could include a hydrolyzed infant formula.*
✓ *When cow's milk protein formula has been introduced in an infant's diet, make sure that regular ingestion (as little as 10 ml daily) is maintained to prevent loss of tolerance.*
✓ *For high-risk infants, encourage the introduction of allergenic foods [e.g., cooked (not raw) egg, peanut] early, not before 4 months of age but since 6 months and during the first year of life, in a safe and developmentally appropriate way, at home. In infants at low risk for food allergy, allergenic foods can also be introduced at around 6 months of age.*
✓ *New foods, including commonly allergenic foods, can be introduced on successive days, with no evidence of harm to this approach.*
✓ *When allergenic foods have been introduced, make sure that ongoing ingestion of age-appropriate serving sizes is regular (i.e., a few times a week), to maintain tolerance.*
✓ *Pre-emptive screening for infant food allergies is not recommended. Families should be counseled that the risk of a severe reaction on the first exposure to an allergen is extremely low.*
✓ *There is currently insufficient evidence to recommend vitamin D, omega 3, or pre- or probiotic supplements to prevent food allergies in infants.*

Finally, the issue of “dietary variety” should also be considered in the prevention of FA. Less food choice in the first year of life is related with an increased risk of asthma and allergies in infancy, whereas greater variety reduces the risk of atopic dermatitis and offer a protection against asthma, allergies, and food sensitization.

The mechanisms of this connection are unclear, but increased exposure to dietary antigens could contribute to this association (12).

Regarding the treatment of FA, the nutritional intervention aims to avoid the allergic reaction by eliminating the food(s), and their derivatives, responsible for the symptoms.

This can lead to nutritional deficiencies, and for this reason, nutritional counseling, and growth monitoring especially in the infant and young child is of paramount importance.

The elimination diet can cause anxiety and stress (worry about feeding, increased responsibility, increased caution), particularly in parents and caregivers and also affect the social activities of the child and his or her family (social restrictions, school, travel, restaurants).

Good nutrition is based on a varied, balanced and individualized diet, and this may be the best model for allergy treatment. Therefore, supporting variety in diet and stimulating gut health are fundamental in the nutritional management of FA. The greatest challenge for those working in the field of FA is to ensure that the quality and quantity of nutrients remain guaranteed, even with one or more exclusions (13).

### 3.2. Dietary management: the dietitian's role

An early diagnosis and appropriate care of food allergy are necessary to allow a good quality of life and nutritional status of the patient. An allergy-focused diet history assisted by the allergy-specialist dietitian may direct the physician to further diagnostic testing; supporting the final diagnosis dietetic expertise is important to conduct a dietary assessment to ensure appropriate intake of energy and essential nutrients and to provide patient-oriented counselling.

Treatment of FA often involves exclusion of foods that contain nutrients essential for growth and development. Children with FA then have an additional risk of growth retardation, which may depend either on an early onset of disease or on an active disease state (extensive atopic dermatitis, GI forms with malabsorption and inflammation, which also often cause inappetence and early satiety).

Nutritional intervention, having the aim of first and foremost ensuring adequate caloric intake and ensuring that all nutrients in excluded foods are taken from alternative food sources, can be effective in enabling growth recovery and prevention of all problems that may arise in adulthood.

So the allergy-specialist dietitian will formulate ad an elimination diet, with the aim to:

– give the family clear guidance regarding the elimination of the food [native, its derivatives, foods that contain it as an ingredient (known source) or as a hidden source]
– guide the family in reading and interpreting labels
– provide a detailed list of substitute foods
– ensure proper nutritional intakes
– identify any deficits and provide vitamin and/or trace element supplements in that case
– use appropriate supplements, in the case of extremely restrictive diets

If the allergic child is not getting adequate caloric intake, free amino acids are oxidized to produce energy and become unusable for protein synthesis. Protein sources of high biological value also include major allergens (milk, eggs, soy, fish and nuts).

Allergy to cow's milk protein (CMA) is very common in children and requires specific nutritional interventions, in all pediatric age groups. Substitute formulas and other alternative “milks” are available in this case.

The child allergic to wheat protein should avoid all foods containing wheat, barley, spelt, kamut, oats, and rye. Many alternative grains and pseudo-cereals are available for patients with wheat allergy, including rice, corn, cassava, buckwheat, amaranth, millet, quinoa, sorghum, teff. It is important to remember that 20% of wheat-allergic individuals may also react to other types of grains. Therefore, the use of these alternative products should be individualized and based on tolerance as defined by the allergy specialist. For this reason, it is important to warn the patient about gluten-free foods, products that may contain guar and carob, two legumes that are cross-reactive with soy.

The egg protein allergic child must eliminate a food that is important for optimal energy-nutrient ratio, consequently, the diet must be balanced to make up for this deficit.

In individuals with polyallergy to foods, the variety of association of individual allergies makes it impossible to formulate unambiguous guidance valid for every child. The appropriate approach will be agreed between allergist and dietitian according to the issues arising from the nutritional assessment.

The detection of anthropometric indices (weight, length/height and BMI) is the most important step in the nutritional assessment, since growth is a sensitive indicator of adequate caloric and protein intake. In the case of dietary inadequacies, weight, a sensitive indicator of energy intake, is affected earlier than stature. In addition, it is necessary to assess the hematochemical parameters (martial, protein, lipid, calcium-phosphorus balance, vitamin D, parathormone, and zinchemia) and the patient's nutritional “status” through a targeted and detailed nutritional history in order to be able to devise a balanced diet according to the patients' age and sex. It is important for the dietitian to learn about the patient's general dietary history in order to be able to guide the quality of the patient's diet. It will be incumbent to ask general questions focusing on the importance of nutrients, perhaps suggesting the completion of a 2–7 day or 24-h food diary. Nutritional deficiency may relate to an overall poor calorie intake, an incorrect allocation of calories among major nutrients, or just inadequate micronutrient intake. For example, it is common to find failure to meet the carbohydrate requirements of those patients whose diets include the elimination of corn and rice in addition to grain protein or again, lower protein intakes, in diets being devoid of milk, egg or soy protein and legumes (14).

Little information exists on the role of nutrition and the effect of nutritional counseling in the field of food allergies. A qualified dietitian is a key component of the multidisciplinary FA team, as the physician on the one hand does not have the opportunity to devote himself to these aspects of FA management as well, just as he does not have sufficient expertise to make calculations of theoretical requirements and assessment of how well they are met in the individual child's diet. Success in managing the diet of the FA patient depends on the ability of a team that fuses pediatric, allergological, nutritional, and educational expertise to educate the patient and his or her family to avoid allergens and substitute them appropriately to ensure nutritional requirements, and to implement a careful follow-up program to assess growth, nutrition, and tolerance acquisition over time.

All official allergology guidelines (World Allergy Organization, DRACMA, USA guidelines, UK NICE guidelines) recognize the important role of nutrition education. A nutritional assessment conducted by a qualified dietitian for pediatric food allergies is therefore now recognized as essential to ensure dietary adequacy and to support parents in finding suitable alternatives to replace the eliminated nutrients.

A diagnostic elimination diet set for the long term should be monitored as well as well balanced by a dietitian, and if nutritional deficits are identified, these diets should be accompanied by the inclusion of vitamin and mineral supplements.

The presence of the dietitian will be necessary in the following cases:

– multiple elimination diets
– growth deficits or suspected malnutrition
– need to set a diet in the breastfed and already weaned patient (determination of the nutritional status and intake of the baby and the nursing mother, with possible administration of supplements)
– teaching/guidance, in collaboration with the physician, on reading packaged food labels regarding hidden allergens
– assume a role in surveillance and prevention of possible AA-related eating disorder in children and adolescents.

The correct quantitative and qualitative intake of food, the possibility of preventing or controlling various diseases taking into account interindividual variability by acting on diet, the food-psychological well-being relationship, as well as the proper storage and safety of food are central issues in the interest of the European community and the scientific community (13).

The dietitian can contribute to preventing or correcting nutritional deficiency in children with FA by assuming a considerable role due to his or her training that combines various skills: assessing the nutritional characteristics of foods and their modifications induced by technological and biotechnological processes, verifying their correct intake to achieve the recommended levels of nutrients for the maintenance of health status, monitoring and evaluating the bioavailability of nutrients in foods and food supplements and their effects, assessing nutritional status for psycho-physical well-being, and disseminating nutrition education.

### 3.3. Milk protein allergy

Cow's milk allergy (CMA) is one of the most common food allergies in infants and its prevalence is still increasing.

A recent ESPGHAN position paper provides rules on how to manage CMA and indicate the appropriate dietary choices in order to guarantee adequate growth. In the case of breastfed infants, mothers should be supported to continue breastfeeding by avoiding milk, dairy products and all cow's milk sources from their diet. In non-breastfed infants all the cow's milk products should be strictly avoided. They included all cow's formula and foods containing cow's milk protein or other unmodified animal milk proteins (e.g., goat's milk, sheep's milk). In formula-fed infants at low risk of anaphylaxis, extensively hydrolyzed (eHF) infant formula is usually recommended, and an amino acid-based formula as a second choice. In cases of high risk of anaphylaxis, amino acid-based formula is the first choice and eHF formula the second (15).

Current guidelines and recommendations, which are based on defined research criteria, remain neutral and do not make recommendations for or against the use of partially or extensively hydrolyzed formulas to prevent IgE-mediated food allergies in children, but do recommend that infants should not be given intact cow's milk protein during the first week of life. Instead, the benefits of using partially hydrolyzed or extensively hydrolyzed formulas in preventing gastrointestinal disorders have been demonstrated (16).

## 4. Novel treatment strategies

### 4.1. New treatment by targeted nutrition

The high prevalence of FA stimulates research to find new effective strategies for the treatment of FA.

The main therapeutic option for FA is still food allergen avoidance but oral or epicutaneous food immunotherapy has emerged as a possible therapeutic strategy to consider in the management of FA.

Among the new treatment hypothesis, those of vitamin D and Omega-3 long-chain polyunsaturated fatty acids have emerged.

The association between vitamin D and allergic disease development has been proposed and different studies reported an association between low serum vitamin D levels and the development of allergic diseases (16). Nevertheless, this association may not be causal. A recent systematic review concludes that supplementation of vitamin D for pregnant women, breastfeeding women, and infants do not have an effect in primary prevention of allergic diseases (17). Essential fatty acids and their metabolites are considered important regulators by impacting on immunological reactions. In particular, byproducts derived from omega-3 polyunsaturated fatty acid are anti-inflammatory and/or pro-resolving lipid mediators. In contrast, eicosanods originated from omega-6 LCPUFAs have pro-inflammatory and pro-allergic activity. Because of their competition for the same enzymatic pathways, an increase intake with the diet of omega-3 PUFA, togheter with a decrease of omega-6 PUFA assumption, might theoretically reduce the onset of human immunologic conditions, including allergies, thanks to the replacement of EPA and DHA derivated AA in the membranes of inflammatory cells.

Regarding the treatment of children with CMA, specific hypoallergenic formulas have been reformulated with the objective of modulating the gut microbiome and early immune responses through the addition of lactose and probiotics.

Of course, these hypotheses need further research to improve and refine FA prevention strategies and make them more effective at the population level. 4.2. The hypothesis of Vitamin D and Omega-3 Long-Chain Polyunsaturated Fatty Acids.

An association between low vitamin D levels and FA has been described by several authors (18–20).

Among them, an Australian study showed the association of insufficient serum level of vitamin D (<50 nmol/L) with a significant increased risk of peanut and/or egg allergy. This result agrees with the remark that FA and eczema are more common in regions with a lower sun exposure and poorer levels of skin-derived vitamin D (21).

Thus, inadequate levels of vitamin D in the first year of life may be a risk factor for the development of FA, while a safe supplementation could optimize infant immune health reducing the risk of allergic diseases (not only FA but also allergic respiratory diseases).

On the other hand, a recent cohort study (LINA study) showed that high serum levels of vitamin D during pregnancy and at birth were associated with an increased risk of FA (22, 23).

They hypothesized that high doses of vitamin D could inhibit the maturation of dendritic cells and hinder the development of T-helper 1 responses, increasing the risk of allergic sensitization.

So we have to pay attention because while adequate levels of vitamin D may confer a protective effect, insufficiency and excessive supplementation could rise the risk of allergic diseases in children (18, 19).

A prospective randomized trial, that is still ongoing, is evaluating the role of postnatal vitamin D supplementation as a preventive strategy against IgE-mediated FA, eczema, and inferior respiratory tract infections (24).

Several studies have demonstrated the protective role of polyunsaturated fatty against the development of allergies in infants, if maternal diets are rich in omega-3 long-chain polyunsaturated fatty acids (LCPUFA) during pregnancy (25).

Dunstan et al. showed that supplementation during pregnancy with docosahexaenoic acid and eicosapentaenoic acid increases LCPUFA concentrations in breast milk (26).

On maternal fish oil supplementation during pregnancy, a randomized clinical trial demonstrated a significant decrease in concentrations of IL-4 and IL-13 (Th-2 cytokines) in cord blood and an increase in oral tolerance-inducing TGF-beta levels (27).

In infants with a family history of atopy, the effect of high-dose fish oil supplementation in pregnant mothers (from the 21st week of gestation until delivery) had showed a significantly lower rates of atopic eczema and egg sensitization (28).

The same authors, in another study, randomized high-risk infants and administered daily docosahexaenoic acid (280 mg) plus eicosapentaenoic acid (110 mg) or olive oil (control) from birth to 6 months of age. In this study, no differences in allergic sensitization, eczema, asthma or FA have been observed between the two groups of children.

It can be concluded that some nutrients supplementation during pregnancy can reduce the risk of atopic eczema and food sensitization, but the same supplementation in infants after birth can be ineffective (29).

## 5. Personalized nutrition

An emerging area of study is the long-term effects on eating patterns and food preferences of children with fussy eating due to the exclusion of cow's milk and other foods during infancy (30).

Even if there is still low literature on this aspect, food allergies inevitably affect the quality of life, because of the worry and the awareness required to avoid ingestion. It is frequent that children and adolescents with FA may develop anxiety or, on the contrary, may show challenging behaviors (13).

The main goal for dietary management is to elaborate a complete, individualized treatment plan, which has to consider the medical condition of the patient, food avoidance strategies, healthy eating, nutritional needs and the patient's family life. The dietitian should also take into account the immunological mechanisms, the different clinical presentation of FA and individual tolerance levels of allergens to instruct correctly parents/caregivers and patients.

In practice, the dietitian must give healthy eating advice on an adequate and balanced diet, advising the proper substitutes for the excluded foods, to reduce as much as possible the impact on quality of life. Doing so, a relief of patients' symptoms occurs, by avoidance of allergenic foods. The dietitian must also fix macro and micronutrient requirements with a personalized diet to guarantee normal growth and development for age and gender. The importance of suggesting alternative and safe foods to replace the avoided foods in the context of a diversified diet, need of verifying if all nutritional requirements are satisfied (e.g calcium and vitamin D for cow's milk allergic children).The dietitian has also the role of teaching how to correctly interpret food labels in order to reduce as much as possible the inadvertent exposure to the culprit foods and to prevent the patient from an excessive useless avoidance of foods.

All children with FA should be reassessed periodically as appropriate follow-up allows to asses dietary compliance and ensure optimal growth.

One of the main objectives of personalized nutrition in children with FA is to prevent unnecessary dietary restrictions. The dietitian should also understand the immunological mechanisms, the different clinical presentations and tolerance levels of food allergies. To date, a large percentage of children with CMA and egg allergy are able to tolerate the heat- treated allergen. Cooking has the effect of destroying the conformational epitopes of thermolabile proteins, thus increasing the effect of enzymatic digestion and reducing intestinal absorption and stimulation of basophils. Baked eggs and baked milk are easier to tolerate for some allergic children, thanks to the so-called matrix effect. Indeed, the interactions between milk proteins and some food matrix components during heating seem to limit the accessibility of peptides to the immune system, thus playing an important role in reducing allergenicity (30).

## 6. Discussion

**Figure 1:**
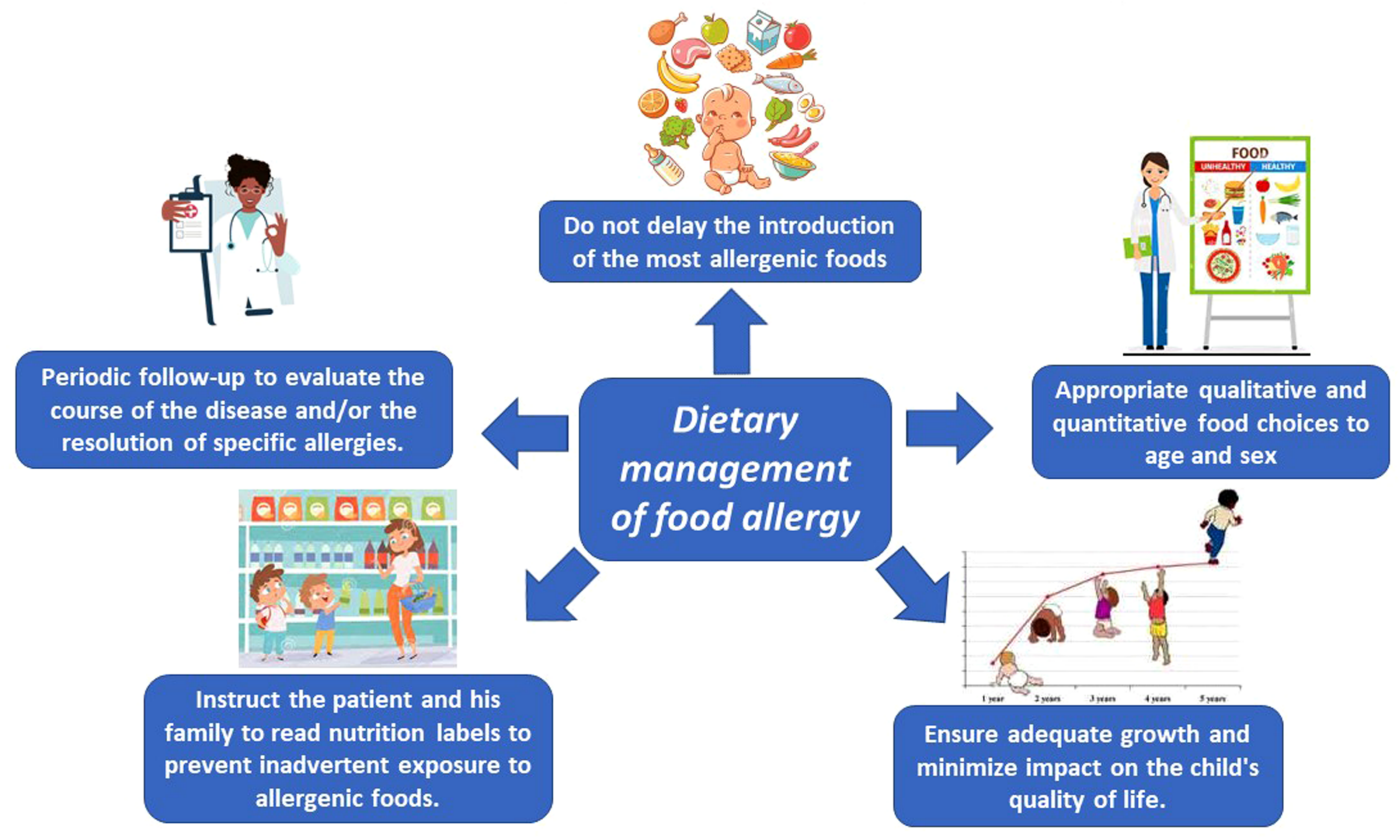
Factors to be considered in making a correct dietary management of food allergy.

The key words for ideal management of FA are nutritional counseling and growth monitoring. Figure 1 shows the factors to be considered in making correct dietary management of FA.

The Dietitian, taking all this information into account, can devise an individualized nutritional plan that best suits the patient's needs and schedule periodic reassessments to evaluate the course of the disease and/or the resolution of specific allergies.

The wider application of the new treatment strategies may have as the most important goals to improve health and quality of life for both patients with FA and their families/caregivers.

To support this new treatment, need further research to improve and refine FA prevention strategies and make them more effective at the population level.
